# Magnetic Resonance Imaging of Transplanted Porcine Neonatal Pancreatic Cell Clusters Labeled with Chitosan-Coated Superparamagnetic Iron Oxide Nanoparticles in Mice

**DOI:** 10.3390/polym13081238

**Published:** 2021-04-11

**Authors:** Jyuhn-Huarng Juang, Jiun-Jie Wang, Chia-Rui Shen, Chen-Yi Chen, Chen-Wei Kao, Chen-Ling Chen, Sung-Han Lin, Shu-Ting Wu, Wan-Chun Li, Zei-Tsan Tsai

**Affiliations:** 1 Division of Endocrinology and Metabolism, Department of Internal Medicine and Center for Tissue Engineering, Chang Gung Memorial Hospital, Taoyuan 33305, Taiwan; Je3474@gmail.com (C.-Y.C.); lian8807111@gmail.com (C.-W.K.); jenny74513@gmail.com (C.-L.C.); 2 Department of Medicine, College of Medicine, Chang Gung University, Taoyuan 33302, Taiwan; 3 Department of Medical Imaging and Radiological Sciences, College of Medicine, Chang Gung University, Taoyuan 33302, Taiwan; jiunjie.wang@gmail.com (J.-J.W.); image.lin@gmail.com (S.-H.L.); 4 Department of Diagnostic Radiology, Chang Gung Memorial Hospital, Keelung 20401, Taiwan; 5 Department of Medical Biotechnology and Laboratory Science, College of Medicine, Chang Gung University, Taoyuan 33302, Taiwan; crshen@mail.cgu.edu.tw (C.-R.S.); proteinwhite@livemail.tw (S.-T.W.); 6 Institute of Oral Biology, School of Dentistry, National Yang Ming Chiao Tung University, Taipei 11221, Taiwan; wcli@ym.edu.tw; 7 Molecular Imaging Center, Chang Gung Memorial Hospital, Taoyuan 33302, Taiwan; zeitsan@ms9.hinet.net

**Keywords:** porcine neonatal pancreatic cell clusters, transplantation, magnetic resonance imaging, chitosan-coated superparamagnetic iron oxide nanoparticles

## Abstract

Neonatal pancreatic cell clusters (NPCCs) are potential tissues for the treatment of diabetes. Different from adult cells, they continuously proliferate and differentiate after transplantation. In this study, we utilized magnetic resonance imaging (MRI) to detect and monitor implanted NPCCs. NPCCs were isolated from one-day-old neonatal pigs, cultured for three days, and then incubated overnight with the contrast agent chitosan-coated superparamagnetic iron oxide (CSPIO) nanoparticles. In vitro, Prussian blue staining and MR scans of CSPIO-labeled NPCCs were performed. In vivo, we transplanted 2000 CSPIO-labeled NPCCs under the kidney capsule of nondiabetic nude mice. Recipients were scanned with 7.0T MRI. Grafts were removed for histology with insulin and Prussian blue staining. After being incubated overnight with CSPIO, NPCCs showed positive iron staining and appeared as dark spots on MR scans. After transplantation of CSPIO-labeled NPCCs, persistent hypointense areas were observed at recipients’ implant sites for up to 54 days. Moreover, histology showed colocalization of the insulin and iron staining in 15-, 51- and 55-day NPCC grafts. Our results indicate that transplanted NPCCs survived and differentiated to β cells after transplantation, and that MRI is a useful tool for the detection and monitoring of CSPIO-labeled NPCC grafts.

## 1. Introduction

Since 2000, human islet transplantation has proven efficacious in curing patients with type 1 diabetes. However, most successful cases need two or more transplants [1,2,3]. To solve the problem of limited pancreas donors, several alternative β-cell sources are currently being explored, particularly xenogeneic islets and pluripotent stem cells [4,5]. Pigs are promising replenishable source of islets. However, adult porcine islets are fragile during isolation [6], and fetal islets have a poor insulin response to glucose [6,7]. In contrast, porcine neonatal pancreatic cells (NPCCs) are easily isolated, and capable of secreting insulin in response to glucose and restoring normoglycemia after transplantation in diabetic mice [8,9,10,11,12], pigs [13], and nonhuman primates [14]. However, they are rather immature and continue differentiation in vitro [8,9,15] and in vivo [9,10,15].

To better understand the fate of islets after transplantation, magnetic resonance imaging (MRI) has been used to detect transplanted islets labeled with dextran-coated superparamagnetic iron oxide (SPIO), such as ferumoxide (Feridex^®^, EndoremTM) and ferucarbotran (Resovist^®^), in mice [16,17,18,19], rats [20,21,22,23,24,25,26,27], baboons [28], and humans [29,30]. However, MRI has not yet been used in tracing transplanted NPCCs which undergo differentiation. Since Feridex^®^ and Resovist^®^ were no longer available after 2008 and 2009, respectively [31], it is crucial to develop new MR contrast agents for cell imaging. Chitosan, the *N*-deacetylated product of chitin, is one of the most abundant polysaccharides in nature. It has been applied to numerous biomedical applications due to its nontoxicity, biocompatibility, and biodegradability [32]. It is particularly interesting in metal nanoparticle synthesis because of its interaction with metal atoms, metal ions, and metal oxide nanoparticles for their stabilization in colloidal suspension. In this regard, it is worth noting that chelation evenly disperses metal oxides throughout the chitosan polymer. Chitosan is therefore a good dispersant for a variety of different nanoparticles, including single-walled carbon nanotubes [33,34]; platinum, gold, and silver nanoparticles [35]; as well as iron oxide nanoparticles. Of note, the primary amines on chitosan are involved in metal ion chelation and nanoparticle immobilization [36,37]. We have developed an in situ coating method to prepare ferrofluids coated with γ-ray-irradiated chitosan [38] and demonstrated that the chitosan-coated SPIO (CSPIO) nanoparticles have potential as a T2 contrast agent in MRI [39]. We also showed that CSPIO-labeled adult mouse islet isografts [40,41] and allografts [40,42] can be safely and effectively imaged by MR for a long period of time. However, it is not known if CSPIO nanoparticles can internalize into NPCCs and be applied for imaging NPCC grafts by MRI. Therefore, in this study, we tested if MRI can be used for the detection and monitoring of transplanted NPCCs labeled with CSPIO nanoparticles. 

## 2. Materials and Methods

### 2.1. Materials

RPMI-1640 medium was purchased from GIBCO BRL (Grand Island, NY, USA). Collagenase type V was from Sigma Immunochemicals (St Louis, MO, USA). Iron(III) chloride hexahydrate (FeCl_3_⋅6H_2_O) and D–mannitol were obtained from Riedel-de Haen (Seelze, Germany). Iron(II) chloride tetrahydrate (FeCl_2_⋅4H_2_O) was from Showa (Minato City, Tokyo, Japan). Ammonium hydroxide solution (25%) was obtained from Fluka (Buchs, Switzerland). Low-molecular-weight chitosan was from Sigma-Aldrich (St. Louis, MO, USA). Chitosan had a molecular weight of 50–190 kDa and a deacetylation degree of 83.3%. Solid chitosan was subjected to Co-60 g-ray irradiation at a dose of 300 kGy prior to use. After irradiation, the molecular weight of chitosan was between 13–16.2 kDa, determined by using a Cannon Ubbelohde four bulb shear dilution viscometer. Deionized water was purged with nitrogen gas for 30 min before use. Polyethylene tubing (PE-50) was purchased from Clay Adams (Parsippany, NJ, USA). Guinea pig anti-swine insulin antibody was purchased from Dako (Carpinteria, CA, USA).

### 2.2. Preparation of CSPIO 

Approximately 0.428 g of irradiated solid chitosan were dissolved in 200 mL 0.5% (*v*/*v*) aqueous acetic acid. To the irradiated chitosan solution, 2 g of FeCl_3_⋅6H_2_O (7.4 mmole) and 0.90 g of FeCl_2_⋅4H_2_O (4.5 mmole) were added to obtain a pale brown solution. Subsequently, 10 mL of 25% ammonium hydroxide solution was rapidly added to the brown solution under sonication at 50 °C. The mixture was treated for a further 40 min with sonication. The black precipitate was isolated with the aid of a magnet and decantation or centrifugation at 3500 rpm for 10 min. Subsequently, it was washed with water at least three times until there was no AgCl cloud. The washed black precipitate was converted to ferrofluid of CSPIO particles—an aqueous solution of complex chitosan with SPIO—when dispersed in 60 mL water (total volume) at a pH of 2.0, obtained by the addition of 2 N HCl, 3.48 g of mannitol, and 2 mL of lactic acid. 

### 2.3. Animals 

One-day-old pigs of either sex were purchased from a local slaughterhouse. Male athymic nude Balb/c mice, aged 8 to 12 weeks, were purchased from the National Laboratory Animal Center and used as recipients of the NPCCs. The animal experimental protocol was approved by the Institutional Animal Care and Use Committee of Chang Gung Memorial Hospital.

### 2.4. Preparation and Culture of NPCCs

Each neonatal pig pancreas was cut into fragments from ~ 1 to 2 mm^3^, then digested by collagenase type V in a water bath at 37 °C. The digest was filtered, washed, then placed in RPMI-1640 medium and maintained at 37 °C (5% CO_2_, 95% air) in humidified air [12]. After 3 days of culture, NPCCs were incubated overnight with CSPIO nanoparticles (concentration of 10.08 μg/mL) before in vitro studies and transplantation. 

### 2.5. Uptake of CSPIO Nanoparticles by NPCCs

NPCCs were incubated overnight with CSPIO nanoparticles, and the intracellular iron content was then examined by Prussian blue staining. NPCCs were washed with PBS to remove excess iron particles and then fixed in 4 vol% formaldehyde solution for 30 min. After fixation, the cells were stained for the presence of intracellular iron with freshly prepared potassium ferrocyanate solution (mixture of equal volume of 4 wt% potassium ferrocyanate with 4 vol% hydrochloric acid) for 30 min. After washing with distilled water, the cells were examined using a microscope to determine the labeling efficiency. Cells with intracellular blue particles were considered labeled.

### 2.6. In Vitro MR Scanning

MR imaging was performed on a 7.0 T MRI system (Clinscan, Bruker, Ettlingen, Germany). Seven hundred NPCCs were incubated overnight with CSPIO nanoparticles for 24 h at 37 °C and washed three times in PBS. All samples were scanned by using a fast gradient-recalled echo pulse sequence (repetition time (TR)/echo time (TE) = 3000 msec/70 ms). The contrast enhancement was calculated by the following equation: percentage of enhancement (%) = (SIpost−SIpre)/SIpre × 100, where SIpost is the signal intensity measured from within the phantom of cells with treatment of the contrast agent, CSPIO. SIpre is the signal intensity from the phantom with cells alone [39].

### 2.7. Transplantation of NPCCs

Two thousand CSPIO-labeled NPCCs were transplanted under the left kidney capsule of each nude mouse. NPCCs were first centrifugated in PE-50 tubing connected to a 200-µL pipette tip. A capsulotomy was performed in the lower pole of the left kidney. The tip of the tubing was advanced under the capsule from the lower pole of the kidney capsule to the upper pole, the final injection site [12].

### 2.8. In Vivo MR Scanning

After transplantation, serial MR was acquired on the same scanner in 3 recipients. MR images were acquired on the same scanner using a surface coil with the following parameters for the gradient-recalled echo sequence: slice thickness = 0.5 mm, TR = 3700 ms, TE = 37 ms. MRI signal intensity of the graft at the left kidney and the mirror area at the right kidney, which was used as a within-subject control, was calculated [40,41,42].

### 2.9. Histological Study of Grafts

NPCC grafts were removed at 15, 51, and 65 days after transplantation. They were fixed in a formalin solution and processed for paraffin embedding and sectioning. Sections of grafts were stained with iron with Prussian blue and for endocrine β-cells with a guinea pig anti-swine insulin antibody.

### 2.10. Statistical Analysis

In vivo MR signal intensity was expressed as the mean and standard deviation (M ± SD). All the statistical analyses were performed in PASW Statistics 21 (released 2012, IBM SPSS Statistics for Windows; Armonk, NY: IBM Corp.). The normality of the distribution of the variable was checked with the Kolmogorov–Smirnov test. For each comparison pair of mean values of the graft at the left kidney and the mirror area at the right kidney, the independent *t*-test was performed if both samples passed the normality test. The Mann–Whitney U test (Wilcoxon test) was performed if any one sample of the comparison pair failed with the normality test. A *p*-value < 0.05 was considered significant.

## 3. Results and Discussion

### 3.1. Uptake of CSPIO Nanoparticles by NPCCs

**Figure 1:**
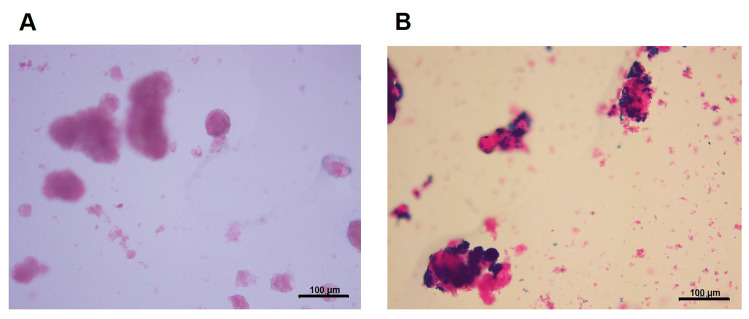
Uptake of chitosan-coated superparamagnetic iron oxide (CSPIO) nanoparticles by NPCCs. NPCCs were incubated overnight without (**A**) or with (**B**) CSPIO nanoparticles. The intracellular iron content was examined by Prussian blue staining.

To track NPCCs in vivo by MRI, it is essential to demonstrate the cell uptake of contrast agent with positive images on MR scans. We have developed and characterized the MR contrast agent, CSPIO nanoparticles, which have a z-average diameter of 87.2 nm, a polydispersity index (PDI) of 0.251, a zeta potential of 47.9 mV, and an iron concentration of 10.4 mg Fe/mL [38]. They do not affect islet viability and insulin secretion [40] and have been used for long-term tracking of islet isografts [40,41] and allografts [40,42]. To examine cellular uptake of CSPIO nanoparticles, NPCCs were incubated overnight with CSPIO nanoparticles, and the intracellular iron content was then examined by Prussian blue staining. Figure 1A shows the absence of blue stain in the NPCCs without CSPIO loading. In contrast, blue spots were located in the cytoplasm of some CSPIO-loaded NPCCs (Figure 1B), indicating that CSPIO nanoparticles were taken up by these cells. These findings are consistent with our previous observation that CSPIO could be introduced into cells, including two pancreatic β-cell lines, NIT-1 and β-TC6 [39]. It is well recognized that cationic chitosan can strongly bind with anionic cell surface, and subsequently enhances nanoparticle internalization via endocytosis [43,44]. Hence, in this study, coating SPIO with chitosan further promoted NPCC uptake of nanoparticles. 

### 3.2. In Vitro MR Image of NPCCs

**Figure 2:**
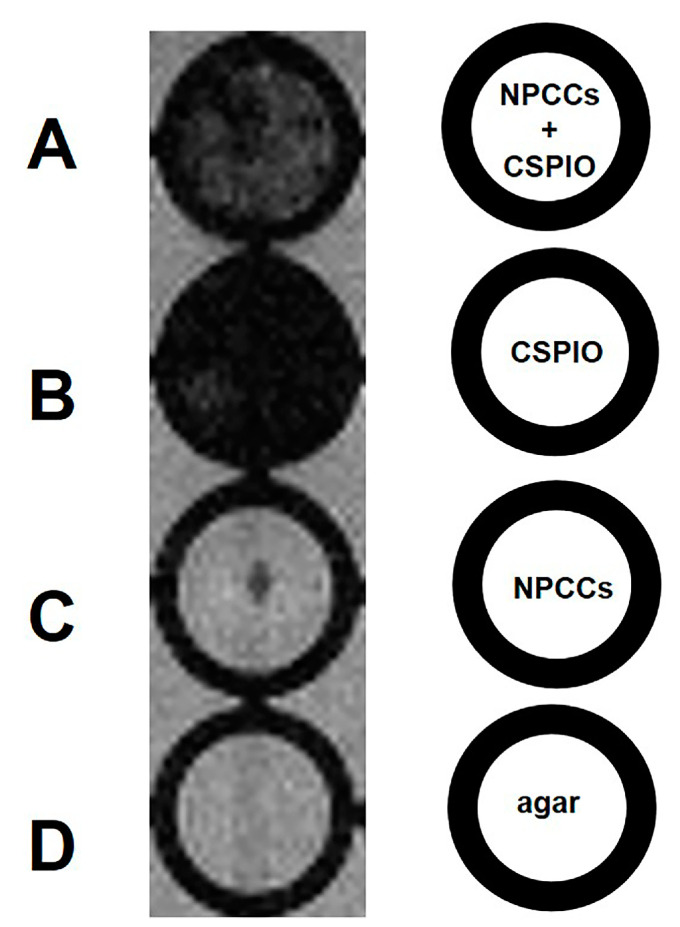
In vitro MRI of 700 NPCCs incubated overnight with (**A**) and without (**C**) chitosan-coated superparamagnetic iron oxide (CSPIO) nanoparticles. CSPIO (**B**) was used as a positive control and agar (**D**) was used as a negative control. All were scanned by a 7.0 T MRI system. In contrast to unlabeled NPCCs (**C**), CSPIO-loaded NPCCs (**A**) appeared as dark spots.

We then perform in vitro 7.0 T MRI on 700 NPCCs, incubating overnight with and without CSPIO nanoparticles (Figure 2A,C). CSPIO (B) was used as a positive control, and agar (D) was used as a negative control. As expected, there was a background image of the agar (Figure 2D) and a completely dark image of CSPIO nanoparticles (Figure 2B). In contrast to a background image in NPCCs without CSPIO labeling (Figure 2C), CSPIO-loaded NPCCs appeared as dark spots (Figure 2A), corresponding to the locations of loaded cells [39]. Visualization of CSPIO-labeled NPCCs by in vitro MRI is fundamental for detecting them by MRI after transplantation. 

### 3.3. In Vivo MR Images of NPCCs after Transplantation

**Figure 3:**
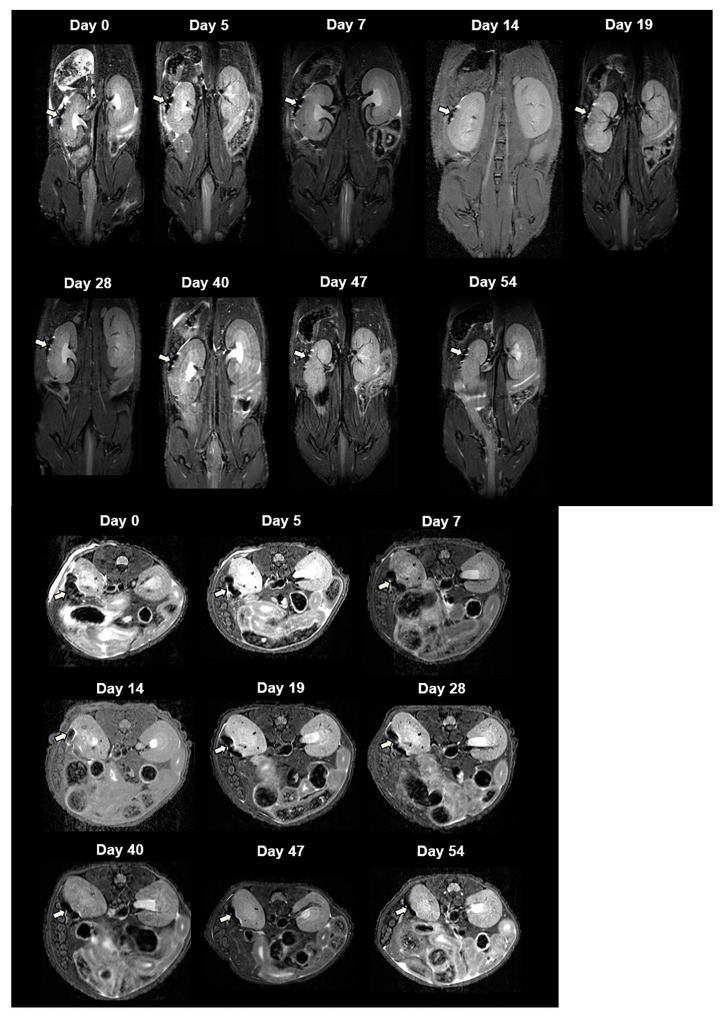
In vivo MR image of NPCCs after transplantation. Two thousand CSPIO-labeled NPCCs were transplanted under the left kidney capsule of a nude mouse. The recipient was scanned by a 7.0 T MRI system with coronal (upper panel) and transverse (lower panel) sections. The graft of CSPIO-labeled NPCCs was indicated by arrows.

**Figure 4:**
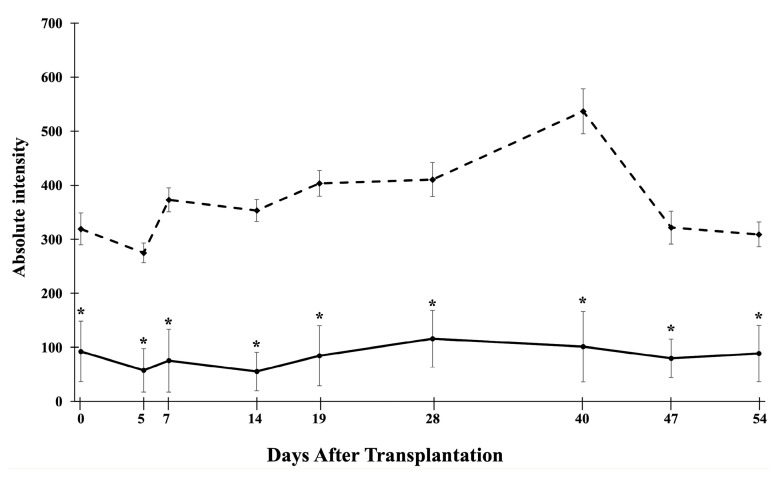
Time course of the MR signal intensity of the graft on the left kidney (solid line), and the mirror area on the right kidney (dash line). * *p* = 0.000.

For in vivo MRI, we transplanted 2000 CSPIO-labeled NPCCs under the left kidney capsule of each nude mouse. Three recipients were scanned by a 7.0 T MRI system for up to 54 days. As shown in Figure 3, the graft of CSPIO-labeled NPCCs (indicated by arrows) was visualized as a distinct hypointense area on MR images located at the implantation site between day 0 and 54. This is expected, as we previously visualized CSPIO-labeled islets under mouse kidney capsules on MR scans as persistent hypointense areas after syngeneic and allogeneic transplantation [40,41,42]. The quantification analysis revealed that the MR signal intensity of the graft on the left kidney was significantly reduced compared to the mirror area on the right kidney at all time points (*p* = 0.000) (Figure 4). Previously, we observed that the MR signal loss was 20% lower in 200 CSPIO-labeled islet isografts than that of unlabeled islet isografts, and this difference persisted for 6 weeks [40]. In this study, 2000 CSPIO-labeled NPCC grafts made a persistent 60–80% reduction of MR signal for 54 days as compared to the same area in the contralateral kidney. To the best of our knowledge, we are the first to use MRI for the detection and monitoring of NPCC grafts.

### 3.4. Histological Studies of NPCC Grafts

**Figure 5:**
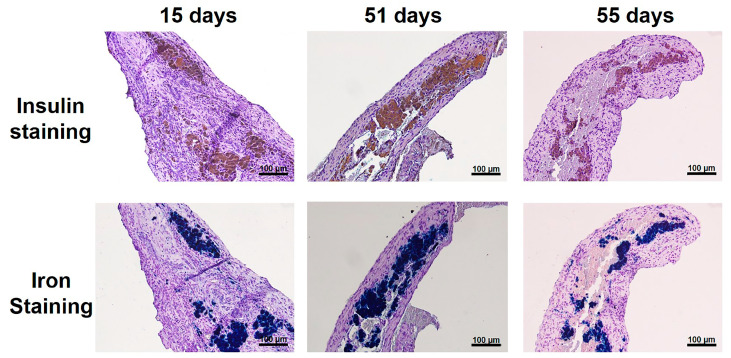
NPCC grafts at 15, 51, and 55 days after transplantation. The graft was stained with insulin (upper panel, brown color) and Prussian blue (lower panel, blue color), which were colocalized.

NPCC Grafts were removed from recipients at 15, 51, and 55 days after transplantation. To investigate the graft microscopically, we used an insulin antibody to stain NPCCs and Prussian stain to stain iron. As shown in Figure 5, all grafts were stained positive for insulin and iron, which were colocalized. These results indicate that transplanted NPCCs not only survived, but also differentiated to β cells after transplantation.

## 4. Conclusions

We have developed an in situ coating method to prepare ferrofluids coated with γ-ray-irradiated chitosan, CSPIO nanoparticles [38], and demonstrated their potential to be a T2 contrast agent in MRI [34]. We also showed that CSPIO-labeled islet isografts [40,41] and allografts [40,42] can be safely and effectively imaged by MR for a long period of time. In this study, our results indicate that transplanted NPCCs survived and differentiated to β cells after transplantation, and that MRI is a useful tool for the detection and monitoring of transplanted NPCCs labeled with CSPIO nanoparticles.
